# Supplementary material for: One Copy Number Variation within the Angiopoietin-1 Gene Is Associated with Leizhou Black Goat Meat Quality
Source: Animals (Basel). 2024 Sep 14;14(18):2682. doi: 10.3390/ani14182682 (PMC11428527; doi:10.3390/ani14182682)
Supplement: Supplementary file 1 [file animals-14-02682-s001.zip › animals-3155390-supplementary.pdf]

Supplementary Materials

Table S1. Primer information

Gene name		5'-3'	Length	Note
ANGPT1	F	GGTGGGGCAATGAGAGGAAA	150	Exonic-CNV1
	R	ACAGGGTGTATGGAGAGCCT		Chr14:24950807-24950937
	F	GTGGTTGGGGAGTTCCAAGT	233	Intronic-CNV2
	R	CCAGTTACTCAGGCTCCACG		Chr14:25041614-25041827
	F	CAAAGAGTAGAGATGGGAAAGGCCA	135	Intronic-CNV3
	R	TTACCCTTACGTCTTCTGTGCC		Chr14:25113621-25113734
MC1R	F	GGGCAGTCCCTTGACAAAGA	129	CNV-qPCR
	R	ATCTCCCCAGCCTCCTCATT		
ANGPT1	F	GAAGGGTCACACTGGGACAG	134	mRNA-qPCR
	R	CAAACCACCAGCCTCCTGTT		
GADPH	F	TGAAGGTCGGTGTGAACGGATTTGG	277	mRNA-qPCR
	R	ACGACATACTCAGCACCAGCATCAC		

Table S2. The copy number spectrum of three CNVs in goat *ANGPT1* gene.

**CNVs**	**Copy Number Spectrum**							
	**1 copy**	**2 copies**	**3 copies**	**4 copies**	**5 copies**	**6 copies**	**7 copies**	**8 copies**
**CNV-1**	0	319	61	37	-	-	-	-
**CNV-2**	31	245	94	46	-	-	-	-
**CNV-3**	53	201	137	17	2	4	1	2

Table S3. The association analysis between the traits and the goat *ANGPT1* CNV-2.

**Traits**	**CNV Types (Mean ± SE)**			***p* values**
	**Loss （1 Copy） (*n* = 31)**	**Normal (2 Copies) (*n* =245)**	**Gain (≥3 Copies) (*n* = 140)**	
body weight (BW, kg)	20.14±0.22	20.39±0.17	21.03±0.16	0.037
Carcass weight (CW, kg)	9.07^{b}±0.18	9.32^{b}±0.21	9.98^{a}±0.15	0.025
shear stress (SS, N)	48.79^{a}±0.28	48.46^{b}±0.12	46.33^{b}±0.21	0.043
